# Emerging Role, Molecular Mechanisms, and Therapeutic Potential of 2-O, 3-O Desulfated Heparin (ODSH) Across Inflammatory and Vascular Diseases

**DOI:** 10.3390/antiox15091179

**Published:** 2026-09-17

**Authors:** Rahul S. Patil, Shweta R. Patil, Joyce N. Gonzales, Alexander D. Verin

**Affiliations:** 1 Vascular Biology Center, Medical College of Georgia, Augusta University, Augusta, GA 30912, USA; shwpatil@augusta.edu (S.R.P.); averin@augusta.edu (A.D.V.); 2 Division of Pulmonary, Critical Care, and Sleep Medicine, Medical College of Georgia, Augusta University, Augusta, GA 30912, USA; jgonzales@augusta.edu

**Keywords:** 2-O, 3-O desulfated heparin, glycosaminoglycans, HMGB1–RAGE–TLR4 axis, endothelial barrier dysfunction, neutrophil elastase, macrophage dysfunction, inflammation, translational therapeutics

## Abstract

Glycosaminoglycans (GAGs) are structurally diverse polysaccharides that regulate inflammation, coagulation, and cellular homeostasis through highly specific sulfation patterns and interactions with cationic proteins. Among GAG derivatives, 2-O, 3-O desulfated heparin (ODSH) is a chemically modified heparin derivative characterized by markedly reduced anticoagulant activity yet preserved affinity for key inflammatory mediators. This review summarizes current knowledge on ODSH structure–function relationships, molecular mechanisms, and therapeutic applications across inflammatory, vascular, and immune-mediated diseases. ODSH modulates several convergent pathogenic processes, including HMGB1-RAGE/TLR4 signaling, neutrophil elastase-mediated tissue injury, PF4-dependent platelet activation, selectin-mediated adhesion, and thrombin-induced endothelial permeability. These actions arise from both direct molecular interactions and indirect sequestration of extracellular mediators, collectively associated with reduced cytokine release, enhanced endothelial barrier stability, and attenuation of inflammation–coagulation crosstalk. Preclinical studies report protective effects in acute lung injury, cystic fibrosis, ischemia–reperfusion injury, traumatic brain injury, thrombocytopenia, and metastatic cancer. Some pilot clinical observations in acute myeloid leukemia and septic peritonitis have reported favorable biological effects. Despite multimodal activity, limitations include incomplete pharmacokinetic characterization, limited comparative studies with other desulfated heparins, and the need for dose–response and safety evaluation in human trials. Overall, available evidence suggests that ODSH may have therapeutic relevance in diseases characterized by excessive inflammation, endothelial dysfunction, and dysregulated host responses. This review integrates current evidence to guide future mechanistic and translational investigation into ODSH and related low-anticoagulant heparin derivatives.

## 1. Introduction

The biological activity of glycosaminoglycans (GAGs) is largely dictated by their sulfation-dependent interactions with inflammatory mediators, coagulation proteins, and growth factors, making this class of polysaccharides important modulators of vascular and immune homeostasis. Heparin, the most highly sulfated member of this family, has been used for more than a century as an anticoagulant; however, accumulating evidence demonstrates that its biological activities extend far beyond antithrombin-mediated inhibition of coagulation factors. Heparin and its derivatives can modulate leukocyte adhesion, neutralize proteases, bind inflammatory mediators, and influence endothelial barrier stability, highlighting their broader relevance in inflammatory and vascular diseases [1,2,3].

Selective chemical modification of heparin has enabled the development of derivatives with reduced anticoagulant activity but preserved or enhanced anti-inflammatory and cytoprotective functions (low-anticoagulant heparins). Among these engineered molecules, 2-O, 3-O desulfated heparin (ODSH) is a chemically modified heparin derivative under active preclinical investigation. Removal of the 2-O and 3-O sulfate groups diminishes antithrombin binding while maintaining affinity for key mediators such as high mobility group box 1 (HMGB1), neutrophil elastase, platelet factor 4 (PF4), and selectins [1,2,4]. This structural refinement allows ODSH to target pathological processes that are not adequately addressed by unfractionated heparin or low-molecular-weight heparins, including HMGB1-RAGE/TLR4 signaling, elastase-driven tissue injury, PF4-dependent platelet activation, and thrombin-induced endothelial permeability [5,6,7].

Over the past two decades, ODSH has been evaluated across diverse disease models characterized by inflammation–coagulation–endothelial dysfunction crosstalk. Preclinical studies have reported protective effects in acute lung injury (ALI) [8,9], cystic fibrosis (CF) [5], ischemia–reperfusion (I/R) injury [10,11], traumatic brain injury (TBI) [12], chemotherapy- and radiation-induced thrombocytopenia [13], acute myeloid leukemia (AML) [14], and metastatic cancer [15,16]. These findings are consistent with multimodal mechanistic activity rather than a single dominant pathway. However, the field also contains unresolved questions, including the relative contribution of direct molecular interactions versus indirect sequestration of extracellular mediators, the extent to which ODSH differs mechanistically from other desulfated heparins, and the translational relevance of dose-dependent effects observed in animal models.

Some initial clinical observations have described favorable biological effects. A pilot study in AML reported improved platelet recovery with ODSH administration [14], and dose escalation work in septic peritonitis demonstrated favorable safety profiles [17]. These findings highlight the need for deeper mechanistic investigation and comparative evaluation across heparin derivatives to clarify structure–activity relationships and guide clinical development.

This review summarizes current evidence on ODSH structure–function relationships, molecular mechanisms, and disease-specific actions, with the aim of situating ODSH within the broader landscape of heparin-based therapeutics. By synthesizing insights from pulmonary biology, immunology, hematology, and vascular medicine, we outline the mechanistic basis for ODSH’s multimodal activity, discuss areas of controversy and divergence, and identify key gaps that must be addressed to advance ODSH toward clinical translation. Overall, available evidence suggests that ODSH may have therapeutic relevance in diseases characterized by excessive inflammation, endothelial dysfunction, and dysregulated host responses.

## 2. Heparin and Its Derivatives: Overview of ODSH and Structural Features

**Figure 1:**
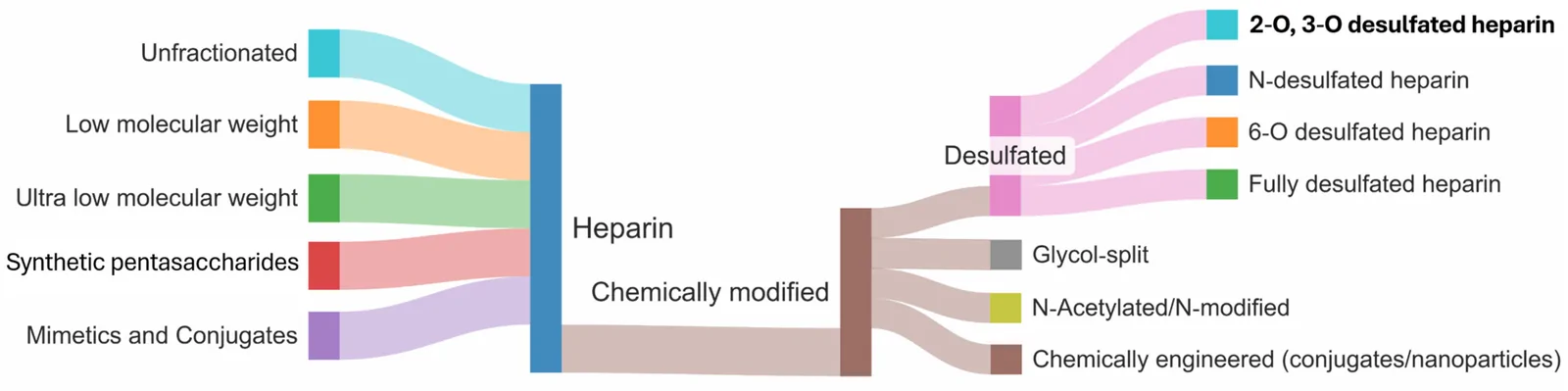
Structural classification of heparin derivatives. Overview of major classes of heparin-based molecules, unfractionated heparin, low-molecular-weight heparin, ultra-low-molecular-weight heparin, synthetic pentasaccharides, mimetics/conjugates, and chemically modified derivatives (figure made at SankeyMATIC.com). Chemically modified derivatives include desulfated heparins, glycol-split heparins, N-acetylated/N-modified derivatives, and other engineered variants. Desulfated heparins can be categorized into 2-O, 3-O desulfated heparin (ODSH, most studied), N-desulfated heparin, 6-O desulfated heparin, and fully desulfated heparin. This classification highlights the structural diversity achieved through selective desulfation and other chemical modifications.

Heparin is a highly sulfated glycosaminoglycan composed of repeating disaccharide units containing iduronic or glucuronic acid linked to glucosamine. The degree and pattern of sulfation at the N-, 2-O-, 3-O-, and 6-O- positions generate substantial structural heterogeneity, which in turn governs heparin’s affinity for antithrombin, coagulation factors, chemokines, growth factors, and extracellular matrix proteins [18]. These sulfation patterns are central to heparin’s biological specificity: the unique antithrombin-binding pentasaccharide sequence is present in only a subset of chains, while other regions mediate interactions with inflammatory mediators, proteases, and adhesion molecules. Figure 1 summarizes the major structural classes of heparin and related derivatives, highlighting how selective modifications alter biological activity.

**Figure 2:**
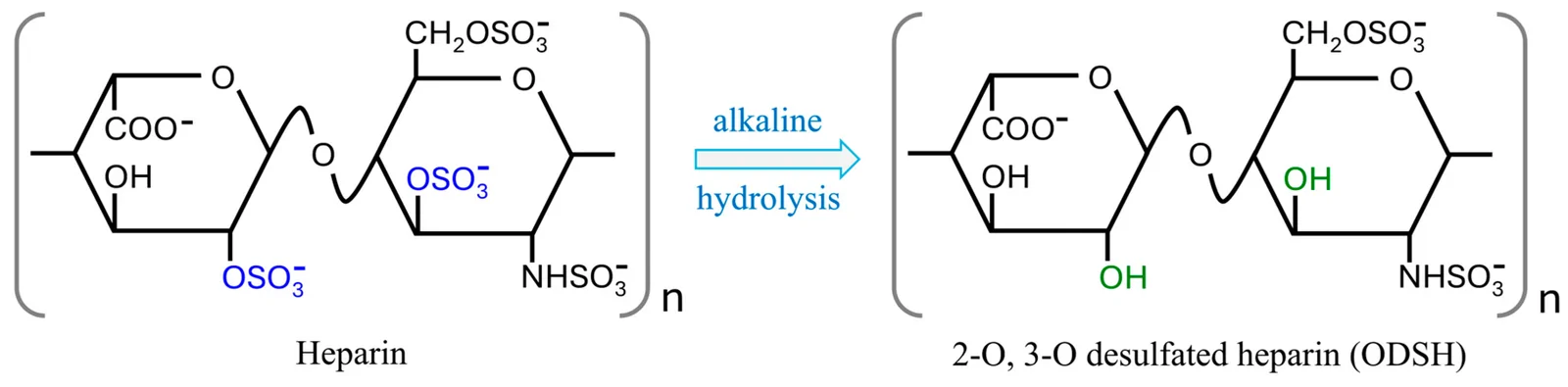
Synthesis of 2-O, 3-O desulfated heparin (ODSH). Schematic representation of ODSH synthesized by controlled alkaline hydrolysis of heparin, which selectively removes 2-O and 3-O sulfate groups while preserving the polysaccharide backbone. This modification reduces anticoagulant activity yet maintains interactions with inflammatory mediators and proteases, thus offering a molecular basis for ODSH’s anti-inflammatory and cytoprotective actions.

Selective desulfation of heparin has enabled the development of derivatives with distinct biological profiles (low-anticoagulant desulfated heparins). While these profiles were not systematically compared, there is a general agreement that removal of specific sulfate groups alters electrostatic interactions and reduces anticoagulant potency while preserving or enhancing anti-inflammatory and cytoprotective functions. 2-O, 3-O desulfated heparin, the most widely studied member of this group [12], is generated through controlled alkaline hydrolysis that selectively removes the 2-O and 3-O sulfate groups from iduronic and glucosamine residues [1,4]. This modification markedly diminishes antithrombin binding and anti-Xa/anti-IIa activity yet maintains high negative charge density and affinity for several cationic proteins central to inflammation and vascular injury. Figure 2 illustrates the controlled alkaline hydrolysis of heparin that removes 2-O and 3-O sulfate groups to produce ODSH.

ODSH retains the ability to bind HMGB1, thereby interfering with HMGB1-RAGE and HMGB1-TLR4 signaling; neutrophil elastase, a major driver of proteolytic tissue injury; PF4, the key mediator of heparin-induced thrombocytopenia; selectins, which regulate leukocyte adhesion and trafficking; and mediators of thrombin-induced endothelial permeability [2,6,7]. These preserved interactions combined with drastically reduced anticoagulant activity provide the mechanistic basis for ODSH’s multimodal anti-inflammatory and barrier-protective effects.

Heparin and its derivatives have long been recognized for their ability to modulate leukocyte–endothelial interactions. Unfractionated heparin reduces peripheral blood mononuclear cell adhesion to activated endothelium [19], and desulfated heparins (including ODSH) exhibit similar or enhanced inhibitory effects on selectin-mediated adhesion [3,20,21,22]. Beyond ODSH, other desulfated derivatives such as N-desulfated, 6-O desulfated, and fully desulfated heparins have been evaluated for specific biological activities [23,24]. However, ODSH remains the most extensively studied low-anticoagulant derivative, with the broadest mechanistic and therapeutic profile across inflammatory, vascular, and immune-mediated diseases.

## 3. Pathophysiology of Inflammatory and Vascular Diseases and ODSH Intervention

A wide range of acute and chronic diseases share convergent pathological features, including excessive inflammation, dysregulated coagulation, endothelial barrier disruption, and maladaptive immune responses. These processes reinforce one another through inflammation–coagulation crosstalk, creating a self-amplifying cycle of tissue injury. Therapeutic strategies that target only one component of this axis often fail to achieve meaningful clinical benefit. The multimodal activity of ODSH positions it as a candidate capable of intervening at several mechanistic nodes simultaneously.

ODSH has been evaluated across diverse pathological states, each characterized by distinct but overlapping pro-inflammatory mechanisms. Below, we summarize key disease contexts in which ODSH has demonstrated preclinical benefit, emphasizing shared pathogenic pathways.

### 3.1. Acute Lung Injury and Acute Respiratory Distress Syndrome

ALI and its severe form, ARDS, are driven by overwhelming inflammation, neutrophil activation, protease release, and endothelial barrier failure [25,26,27]. Endothelial barrier disruption is further influenced by cytoskeletal regulation, mediated by covalent protein modifications (i.e., phosphorylation, acetylation) and junctional stability [25,26]. Lipopolysaccharide (LPS) activates TLR4–MyD88–NF-κB signaling, leading to cytokine release, vascular leakage, and impaired gas exchange. This interplay is also shaped by HDAC-dependent endothelial responses [26] and purinergic signaling pathways that regulate permeability [27]. Current therapies remain largely supportive, with no approved pharmacologic agents that directly target the inflammatory–coagulant interface.

ODSH reduces thrombin-induced endothelial permeability [7], attenuates LPS-driven NF-κB activation [9], and preserves vascular endothelial cadherin (VE-cadherin) junctional integrity [8], consistent with established mechanisms of adherens junction regulation [28,29]. These effects translate into reduced edema, improved oxygenation, and attenuated lung injury in preclinical ALI models.

### 3.2. Cystic Fibrosis and Neutrophil-Dominant Lung Disease

CF is characterized by chronic neutrophilic inflammation, excessive neutrophil elastase activity, and impaired macrophage function. Neutrophil elastase drives mucus hypersecretion, airway remodeling, and progressive lung damage [30,31]. Although CF transmembrane conductance regulator modulators have transformed disease management, they do not fully resolve neutrophil-driven inflammation.

ODSH restores macrophage function compromised by HMGB1 [5], inhibits neutrophil elastase activity [32], and reduces elastase-induced HMGB1 secretion [6], consistent with the central role of HMGB1 in amplifying inflammatory signaling [33,34]. These actions address central inflammatory drivers in CF and neutrophil-dominant lung diseases.

### 3.3. Thrombocytopenia and Chemotherapy-Induced Myelosuppression

Chemotherapy and radiation frequently cause thrombocytopenia, increasing bleeding risk and limiting treatment intensity. Current management relies on platelet transfusions, which carry risks of alloimmunization and infection.

ODSH mitigates thrombocytopenia and accelerates platelet recovery in murine models and some clinical studies [13,14]. These effects are attributed to disruption of PF4–heparin complexes [35,36] and blockade of heparin-induced thrombocytopenia (HIT) antibody-mediated platelet activation by desulfated heparins [37], reduced platelet activation, and modulation of inflammatory pathways that impair megakaryopoiesis, consistent with established mechanisms of HIT immunopathogenesis [38,39,40].

### 3.4. Ischemia–Reperfusion Injury

I/R injury involves oxidative stress, leukocyte recruitment, endothelial dysfunction, and microvascular thrombosis. Conventional anticoagulants reduce thrombosis but do not adequately address inflammation-driven tissue injury, and their broader non-anticoagulant actions have been increasingly recognized [41,42].

Low-anticoagulant heparins, including ODSH, reduce myocyte Na^+^ and Ca^2+^ loading, attenuate edema, and improve tissue recovery after cerebral and myocardial I/R injury [10,11], consistent with emerging evidence that non-anticoagulant heparins modulate pyroptosis and HMGB1/RAGE-dependent injury pathways in reperfusion settings [43]. These effects highlight the importance of targeting both inflammatory and vascular components of reperfusion injury.

### 3.5. Traumatic Brain Injury

TBI triggers neuroinflammation, leukocyte infiltration, and blood–brain barrier (BBB) disruption. Early administration of low-anticoagulant desulfated heparin reduces edema, limits leukocyte mobilization, and improves cognitive outcomes in preclinical models [12].

These effects reflect broader principles of endothelial junctional regulation and permeability control [28,29,44], and they parallel emerging work showing that thrombin-driven endothelial activation shapes inflammatory extracellular vesicle profiles in vascular injury [45]. Together, these findings highlight the potential of ODSH and related derivatives to modulate neurovascular responses without increasing bleeding risk.

### 3.6. Cancer and Metastasis

Tumor progression and metastasis involve platelet–tumor cell interactions, selectin-mediated adhesion, and inflammatory signaling. Low-anticoagulant heparins inhibit tumor cell adhesion to P-selectin [16], disrupt platelet–tumor cell interactions [15], and enhance the efficacy of chemotherapeutic agents such as gemcitabine [46].

These processes are closely linked to the leukocyte adhesion cascade and selectin biology [47]. Heparan sulfate glycosaminoglycans also regulate key oncogenic signaling pathways [48], supporting the broader translational relevance of heparin-based derivatives and their interactions with tumor-associated proteins [49]. Non-anticoagulant heparins have demonstrated inhibitory effects on tumor progression and metastasis independent of anticoagulation [50], consistent with ODSH’s selectin-mediated anti-adhesive activity. Together, these findings support the potential of ODSH and related derivatives as adjuncts in cancer therapy.

### 3.7. Unifying Mechanistic Theme

Many of the diseases discussed here, despite differing in etiology, share core pathological features: excessive inflammation, protease-mediated tissue injury, endothelial barrier dysfunction, dysregulated coagulation, and maladaptive immune responses. These processes reinforce one another through inflammation–coagulation crosstalk, creating a cycle of injury that is difficult to interrupt with single-target therapies.

ODSH acts at several points within this shared axis. Characteristically, it binds primarily to proteins/mediators involved in inflammatory pathways while lacking anticoagulant activity of intact heparin. More than 400 pro-inflammatory mediators possessing heparin-binding sites were identified [41]. While the mechanisms of selectivity to pro-inflammatory factors are not entirely clear, apparently, ODSH targets multiple inflammatory sites [2]. By inhibiting extracellular HMGB1 release and direct extracellular HMGB1 binding, it disrupts HMGB1-RAGE/TLR4 signaling and reduces downstream NF-κB activation. Its inhibition of neutrophil elastase limits proteolytic damage and prevents elastase-induced HMGB1 release. Through disruption of PF4–heparin complexes, ODSH modulates platelet activation and mitigates thrombocytopenia. In parallel, ODSH interferes with selectin-mediated adhesion, reducing leukocyte recruitment, and preserves endothelial barrier integrity by counteracting thrombin-induced permeability.

Together, these coordinated actions position ODSH to target the intersection of inflammation, coagulation, and endothelial dysfunction; a therapeutic space where current treatments remain limited. This mechanistic diversity explains its multifactorial protective effects across diverse conditions, including ALI, CF, I/R injury, TBI, thrombocytopenia, and metastatic cancer.

Overall, available evidence supports a unifying concept: ODSH functions as a multimodal regulator of inflammation–coagulation–endothelial barrier crosstalk, providing a mechanistic rationale for its broad therapeutic potential in diseases marked by dysregulated host responses.

**Figure 3:**
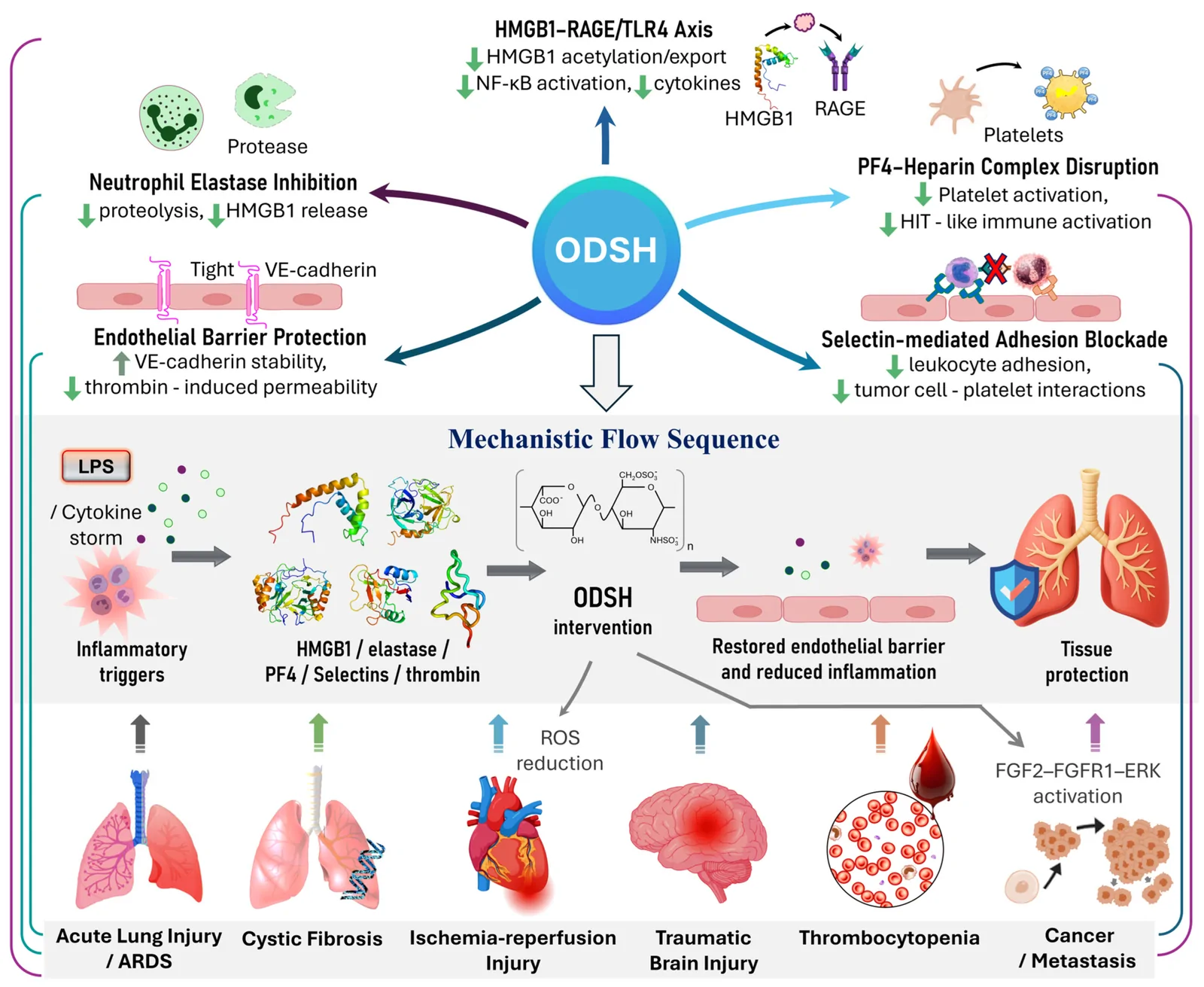
Integrated mechanistic schematic illustrating the multimodal actions of 2-O, 3-O desulfated heparin (ODSH). The central hub shows ODSH interacting with key inflammatory and vascular pathways, including HMGB1–RAGE/TLR4 signaling, neutrophil elastase activity, PF4–heparin complex formation, selectin-mediated adhesion, and thrombin-induced endothelial permeability. The mechanistic flow demonstrates how ODSH intervenes at multiple nodes to reduce inflammation, proteolysis, platelet activation, and vascular leakage. Disease-specific panels highlight the protective effects of ODSH across acute lung injury, cystic fibrosis, ischemia–reperfusion injury, traumatic brain injury, thrombocytopenia, and metastatic cancer. ↓ decrease; ↑ increase.

Figure 3 summarizes the integrated mechanistic schematic on how ODSH targets multiple inflammatory and vascular pathways to achieve tissue protection across diverse disease contexts.

## 4. Mechanisms of ODSH Actions

ODSH exerts its biological effects through a coordinated set of anti-inflammatory, cytoprotective, and immunomodulatory mechanisms. These actions arise from its preserved ability to bind cationic inflammatory mediators and proteases while exhibiting minimal anticoagulant activity. Together, these properties allow ODSH to modulate several interconnected pathways that contribute to inflammation–coagulation–endothelial dysfunction interplay. Table 1 summarizes its structural features and mechanistic profile.

### 4.1. HMGB1-RAGE/TLR4 Axis: Direct Interference with Alarmin-Driven Signaling

HMGB1 is a central alarmin released during tissue injury and infection. It activates inflammatory signaling through RAGE and TLR4, amplifying cytokine production and leukocyte recruitment [33,34]. Extracellular HMGB1 also contributes to systemic inflammation in sepsis and other acute inflammatory states [61,62]. ODSH limits HMGB1 secretion by inhibiting p300 acetyltransferase activity, thereby reducing HMGB1 acetylation and subsequent nucleocytoplasmic translocation and extracellular release. In addition, ODSH directly binds extracellular HMGB1, thus disrupting its interaction with both receptors, thereby reducing downstream NF-κB activation and cytokine release [2,5,53]. This mechanism plays an important role in ODSH-mediated protection in lung injury, CF, and other inflammatory conditions. Consistently, other non-anticoagulant heparins such as N-acetylheparins also attenuate HMGB1–RAGE signaling in I/R injury [63], reinforcing the broader relevance of HMGB1 inhibition.

### 4.2. Neutrophil Elastase: Inhibition of Protease-Driven Tissue Injury

Neutrophil elastase is a major driver of proteolytic tissue destruction, mucus hypersecretion, and impaired host defense in inflammatory lung diseases. Its activity contributes to epithelial injury, extracellular matrix degradation, and amplification of neutrophil-dominant inflammation [30,31]. ODSH directly inhibits elastase activity and prevents elastase-induced HMGB1 release [6,32], reducing both protease-mediated injury and secondary alarmin signaling. These actions are relevant in conditions where excessive serine protease activity shapes disease progression, including CF and ALI [64,65].

### 4.3. Endothelial Barrier Protection: Mitigation of Thrombin- and TLR4-Dependent Permeability

Endothelial barrier dysfunction is a hallmark of ALI, ARDS, sepsis, and other inflammatory vascular disorders. Thrombin and TLR4 signaling promote cytoskeletal contraction, junctional disassembly, and increased permeability. ODSH reduces thrombin-induced permeability through protease-activated receptor (PAR)-dependent mechanisms [7] and modulates TLR4–MyD88–NF-κB signaling to preserve junctional integrity in LPS-induced lung injury [9]. ODSH also helps to preserve VE-cadherin-dependent adherens junctions [8]. These actions align with established mechanisms regulating endothelial permeability, including junctional protein interactions and cytoskeletal tension [28,29,44]. Thrombin-mediated endothelial activation also promotes the release of inflammatory extracellular vesicles, contributing to vascular injury [45]. These actions stabilize VE-cadherin-dependent adherens junctions, reduce edema, and improve oxygenation in preclinical models.

### 4.4. PF4–Heparin Complexes: Disruption of HIT-Associated Multimolecular Assembly

HIT arises from PF4–heparin complex formation and subsequent antibody-mediated platelet activation. ODSH disrupts PF4–heparin multimolecular complexes and reduces platelet activation induced by HIT antibodies [35,37]. This mechanism not only lowers HIT risk but also contributes to improved platelet recovery in chemotherapy-induced thrombocytopenia, a process linked to PF4-dependent immune activation in HIT [38,39,40].

### 4.5. Selectin-Mediated Adhesion: Modulation of Leukocyte and Tumor Cell Interactions

Selectins regulate leukocyte adhesion, endothelial activation, and tumor cell–platelet interactions. Desulfated heparins including ODSH block E-, L-, and P-selectin interactions, reducing leukocyte–endothelial adhesion and tumor cell adhesion [16,20,66]. ODSH potently inhibits E-selectin-mediated adhesion and anti-inflammatory activity [21,22]. These effects support ODSH’s role in inflammation control and metastasis suppression and are consistent with established selectin biology [47,67,68].

### 4.6. Coagulation–Inflammation Interactions: TFPIα and Prothrombinase Regulation

Although ODSH has minimal anticoagulant activity, it retains the ability to influence coagulation–inflammation interplay. ODSH interacts with tissue factor pathway inhibitor-α (TFPIα) and related regulators [55], subtly modulating prothrombinase activity without increasing bleeding risk. Desulfated heparins exhibit reduced anticoagulant activity due to altered TFPIα–prothrombinase regulation [56], providing a mechanistic basis for their safety in trauma, TBI, and sepsis. These interactions are relevant because TFPIα is a key inhibitor of the TF–FVIIa–FXa complex, and changes in TFPIα availability directly affect thrombin generation and downstream inflammatory signaling [43].

### 4.7. Host Defense and Bacterial Clearance: Restoration of Macrophage Function

In CF and pneumonia models, ODSH restores macrophage phagocytic function compromised by HMGB1 and improves bacterial clearance [5,69]. This immune-restorative effect distinguishes ODSH from conventional anti-inflammatory agents that may suppress host defense, highlighting its relevance in infectious and neutrophil-dominant lung diseases. HMGB1 is known to impair macrophage phagocytosis and bacterial killing [33,34,61,62], providing mechanistic support for ODSH’s ability to reverse HMGB1-mediated immune dysfunction. Glycosaminoglycans contribute to bacterial adherence to lung epithelial cells [70], providing additional context for ODSH’s ability to improve bacterial clearance.

## 5. The Potential Therapeutic Value of ODSH

The structural features of heparin, particularly sulfation pattern, charge density, and domain organization, govern its interactions with coagulation factors, inflammatory mediators, and adhesion molecules. Heparin and heparan sulfate share conserved structural motifs that regulate protein binding and biological activity, further supporting the relevance of sulfation pattern in ODSH function [71]. Selective desulfation reduces anticoagulant potency while preserving key protein-binding functions relevant to inflammation and host defense. Foundational studies on heparin structure–function relationships demonstrate that removal of specific sulfate groups alters affinity for antithrombin and other coagulation regulators [41] while maintaining interactions with chemokines, HMGB1, and selectins [49]. These principles explain why ODSH retains anti-inflammatory and immunomodulatory activity despite minimal anticoagulant function. Structural analyses of desulfated heparins further show that targeted modification preserves binding to inflammatory mediators and proteases [42], supporting their therapeutic potential in lung injury, infection, and trauma. Structural variability among pharmaceutical heparins from different animal sources underscores the importance of defined chemical modification in generating consistent non-anticoagulant derivatives like ODSH [72].

Across diverse disease models, ODSH demonstrates a combination of biological properties that distinguish it from unfractionated heparin and other desulfated derivatives. Its structural modifications confer minimal anticoagulant activity while preserving inhibitory interactions with key mediators of inflammation, coagulation, and endothelial injury. Table 2 summarizes the reported mechanisms and therapeutic effects of ODSH.

### 5.1. Minimal Anticoagulant Activity with Preserved Anti-Inflammatory Function

A defining advantage of ODSH is its markedly reduced anticoagulant activity, which minimizes bleeding risk while retaining affinity for inflammatory mediators. This property is particularly important in conditions such as ALI, TBI, and sepsis, where anticoagulation may exacerbate hemorrhage or impair host defense [1,23]. The ability to modulate inflammation without altering coagulation profiles makes ODSH safer than conventional heparin in high-risk settings.

### 5.2. Multimodal Mechanisms Targeting Central Pathogenic Pathways

ODSH simultaneously modulates several conserved drivers of inflammation and tissue injury: HMGB1–RAGE and HMGB1–TLR4 signaling; neutrophil elastase activity; thrombin-induced endothelial permeability; PF4–heparin complex formation; selectin-mediated adhesion; and HMGB1-compromised macrophage function.

This mechanistic versatility allows ODSH to intervene at multiple nodes within the inflammation–coagulation–endothelial dysfunction axis, addressing complex pathophysiology more effectively than single-target agents.

### 5.3. Favorable Safety Profile in Preclinical and Pilot Clinical Studies

Across multiple preclinical studies, ODSH demonstrates excellent tolerability, even at doses substantially higher than those used for anticoagulation. In murine models, ODSH reduces inflammation and tissue injury without increasing bleeding risk [8,13]. Some clinical observations in AML patients and septic peritonitis in dogs also report favorable safety outcomes [14,17], supporting its translational potential. In particular, a dose escalation trial in dogs with septic peritonitis reveals no adverse effects of intravenous ODSH instillation [17]. However, additional larger randomized studies are required to further evaluate the safety and efficacy of ODSH in treatment of septic peritonitis in dogs and humans.

### 5.4. Restoration of Host Defense Rather than Immunosuppression

Unlike corticosteroids or broad immunosuppressants, ODSH restores macrophage phagocytic function impaired by HMGB1 and enhances bacterial clearance [5]. This immune-restorative effect is particularly valuable in CF, pneumonia, and sepsis, where maintaining host defense is essential.

### 5.5. Compatibility with Existing Therapies and Potential for Synergy

ODSH enhances the efficacy of chemotherapeutic agents such as gemcitabine [46] and reduces platelet activation associated with HIT antibodies [37]. These synergistic interactions support its use as an adjunct therapy in oncology and hematology, expanding its therapeutic relevance beyond inflammatory lung disease.

### 5.6. Broad Therapeutic Window Across Organ Systems

ODSH has demonstrated protective effects in multiple organ systems: lung: ALI, ARDS, CF, pneumonia; bone marrow: chemotherapy-induced thrombocytopenia; brain: TBI, cerebral ischemia–reperfusion; heart: myocardial reperfusion injury; cancer: metastasis and platelet–tumor interactions.

While direct simultaneous evaluation of its efficacy in various organs under different disease states was not performed, this universality suggests its ability to modulate conserved inflammatory and vascular pathways across distinct disease contexts.

### 5.7. Multiple Administration Routes

Data from the literature indicated that ODSH may be introduced intravenously [8], intraperitoneally [5], intratracheally [6] and subcutaneously [2] depending on the targeted disease state. However, systematic comparison of different injection routes for one pathology was not evaluated. Limited information suggested that subcutaneous ODSH administration is more effective compared to heparin in inhibiting selectin-mediated lung metastasis in mice [2]. In addition, recent metanalysis of randomized clinical trials suggested that unfractionated nebulized heparin may improve survival of patients with respiratory failure [78]. However, this potentially prospective method for administration of heparin mimetics for treatment of lung diseases was not described for ODSH. Further studies are required to systematically compare various ODSH administration routes in one disease state.

### 5.8. Integrative Rationale

Collectively, these features position ODSH as a therapeutic candidate for diseases characterized by excessive inflammation, endothelial dysfunction, and dysregulated host responses. Its combination of minimal anticoagulant activity, multimodal mechanisms, favorable safety profile, and compatibility with existing therapies provides a compelling rationale for further translational development.

## 6. Translational Insights and Preliminary Clinical Observations

Although ODSH remains in the early stages of clinical development, several studies provide encouraging evidence of its translational potential across hematologic, infectious, oncologic, pulmonary, neurologic, and cardiovascular indications. These findings reflect the central role of HMGB1 signaling, neutrophil elastase activity, PF4 interactions, and endothelial dysfunction across diverse disease states.

### 6.1. Hematologic Applications: AML and Chemotherapy-Induced Thrombocytopenia

A pilot clinical study in patients receiving intensive therapy for AML reported that ODSH was well tolerated and associated with improved platelet recovery [14]. These observations align with murine studies demonstrating mitigation of chemotherapy- and radiation-induced thrombocytopenia [13]. Mechanistically, ODSH disrupts PF4–heparin complexes and reduces PF4-mediated platelet activation [35,37], providing a rationale for its use in supporting hematologic recovery during cytotoxic therapy.

### 6.2. Sepsis and Septic Peritonitis

In a veterinary dose escalation study, ODSH was well tolerated in dogs with septic peritonitis, with acceptable safety across ascending doses [17]. While preliminary, these findings support further evaluation of ODSH in sepsis, where inflammation, endothelial dysfunction, and coagulopathy converge. ODSH’s ability to modulate HMGB1-TLR4 signaling and stabilize endothelial barrier function provides mechanistic justification for its potential use in human sepsis and septic shock. Heparin derivatives have shown protective effects in sepsis by modulating mitochondrial injury pathways [79], supporting the translational potential of non-anticoagulant heparins in systemic inflammatory disease.

### 6.3. Oncology: Pancreatic Cancer and Metastatic Disease

Short communications demonstrated that in pancreatic cancer models, ODSH enhanced the efficacy of gemcitabine and was evidently beneficial when combined with nab-paclitaxel [46,73]. Low-anticoagulant heparins also suppress metastatic dissemination by inhibiting tumor cell–platelet interactions [15], a mechanism relevant to ODSH given its ability to disrupt PF4-dependent complexes and selectin-mediated adhesion. These findings suggest that ODSH may serve as an adjunct therapy to improve chemotherapeutic response and reduce metastatic potential. However, promising results of preclinical studies on noncoagulant heparin derivatives are not always supported by clinical trials [80]; future, more detailed, large-scale clinical trials are warranted to evaluate clinical efficacy of heparin mimetics in human cancers.

### 6.4. Pulmonary Diseases: Cystic Fibrosis and Pneumonia

In CF models, ODSH restored macrophage phagocytic function compromised by HMGB1 and improved bacterial clearance [5]. In *Pseudomonas aeruginosa* pneumonia, partially desulfated heparin improved survival by enhancing bacterial clearance and reducing lung injury [69]. These results highlight the potential of ODSH in chronic and acute pulmonary infections characterized by neutrophil-dominant inflammation and impaired host defense.

### 6.5. Neurologic and Cardiovascular Injuries

Low-anticoagulant desulfated heparin reduced brain edema and improved cognitive outcomes after TBI [12] and decreased myocardial reperfusion injury by limiting myocyte Na^+^ and Ca^2+^ loading [11]. These findings underscore the versatility of ODSH in conditions involving inflammation-driven tissue injury, endothelial dysfunction, and microvascular thrombosis.

### 6.6. Ongoing and Future Clinical Directions

ODSH is currently being explored in combination regimens for pancreatic cancer [81]. Based on its mechanistic profile, future clinical applications may include: ARDS and sepsis, where HMGB1-TLR4 signaling and pulmonary endothelial barrier dysfunction are central; CF exacerbations and pneumonia, where neutrophil elastase and impaired macrophage function drive pathology; trauma and TBI, where preservation of the BBB is critical; myocardial reperfusion injury, where modulation of ion loading and inflammation may improve outcomes; HIT prevention, through disruption of PF4–heparin complexes; adjunctive cancer therapy, targeting platelet–tumor interactions and enhancing chemotherapy efficacy.

The range of these indications reflects ODSH’s ability to modulate conserved inflammatory and vascular pathways across organ systems.

Low-molecular-weight heparins also exhibit multiple non-anticoagulant activities relevant to inflammation and host defense [82], aligning with ODSH’s functional profile. Nebulized heparin formulations have been explored for pulmonary delivery in lung disease [83], highlighting the feasibility of inhaled routes for ODSH.

## 7. Additional Evidence Supporting the Therapeutic Potential of Desulfated Heparins

Beyond ODSH, several structurally related desulfated heparins have demonstrated anti-inflammatory, anti-angiogenic, and tissue-protective activities that further support the therapeutic rationale for low-anticoagulant heparin derivatives. Early work by Wan et al. showed that N-desulfated heparin, a derivative with markedly reduced anticoagulant activity, potently inhibited leukocyte adhesion and transmigration under physiological shear stress and attenuated neutrophil infiltration in murine peritonitis and rabbit ischemia–reperfusion injury [84]. These findings established that selective desulfation preserves anti-inflammatory functions while minimizing bleeding risk, a principle central to ODSH development.

Subsequent mechanistic studies expanded the biological scope of desulfated heparins. Knelson et al. demonstrated that stromal heparan sulfate proteoglycans and soluble heparin derivatives, including 2-O, 3-O desulfated heparin, promote neuroblast differentiation through enhanced FGF2–FGFR1 signaling and ERK activation, suppressing tumor growth in neuroblastoma models [85]. This work highlights the ability of desulfated heparins to modulate growth factor signaling beyond HMGB1 and elastase pathways, suggesting broader relevance in diseases involving aberrant FGF signaling.

Chemical diversification of desulfated heparins has further expanded their functional repertoire. Ji et al. constructed a library of chemically modified and enzymatically depolymerized heparins, demonstrating that selective removal of N-, 2-O-, or 6-O-sulfate groups profoundly alters substrate specificity for heparinases and generates derivatives with potent anti-apoptotic activity in epithelial injury models [86]. These findings underscore the importance of sulfation pattern engineering, a foundational concept underlying ODSH’s multimodal activity.

Recent ophthalmologic studies provide additional evidence for the anti-angiogenic potential of desulfated heparins. Kniggendorf et al. reported that N-desulfated/N-acetylated heparin inhibited endothelial proliferation, migration, and tube formation and significantly reduced choroidal neovascularization in vivo without inducing hemorrhage [87]. Similarly, Grupenmacher et al. showed that 6-O-desulfated heparin suppresses laser-induced choroidal neovascularization and interferes with FGF2-dependent angiogenic signaling [88]. These studies complement earlier biochemical work by Kariya et al. demonstrating that complete 6-O-desulfation preserves heparin’s ability to stabilize and potentiate FGF2 activity [89], collectively indicating that desulfated heparins retain selective growth factor interactions relevant to vascular remodeling.

Additional mechanistic insight comes from cardiovascular research. Wang et al. showed that 6-O-desulfated heparin attenuates myocardial ischemia–reperfusion injury by suppressing miR 199a 5p, restoring klotho expression, and reducing ROS accumulation [90]. This identifies a previously unrecognized miRNA–klotho axis through which desulfated heparins exert cytoprotective effects, expanding the mechanistic landscape beyond classical HMGB1, elastase, and PF4 pathways.

Finally, emerging drug delivery studies demonstrate that desulfated heparins can be chemically engineered for targeted therapeutic applications. Yang et al. developed a three-component drug delivery system using fully desulfated heparin conjugated to galactose and paclitaxel, achieving selective uptake by hepatocellular carcinoma cells while eliminating anticoagulant activity [91]. This highlights the structural versatility of desulfated heparins and their potential for precision therapeutics.

Collectively, these studies reinforce the central concept that selective desulfation preserves or enhances key anti-inflammatory, anti-angiogenic, and cytoprotective functions of heparin while minimizing anticoagulant activity. The breadth of biological effects observed across N-, 2-O-, 3-O-, and 6-O- desulfated derivatives provides strong external validation for ODSH’s mechanistic profile and supports continued translational development of low-anticoagulant heparin analogs.

## 8. Conclusions and Future Directions

ODSH is emerging as a potential therapeutic candidate across inflammatory, hematologic, pulmonary, neurologic, and oncologic diseases. Its selective removal of 2-O and 3-O sulfate groups markedly reduces anticoagulant activity while preserving interactions with key mediators of inflammation and vascular injury. Through modulation of HMGB1–RAGE/TLR4 signaling, inhibition of neutrophil elastase, stabilization of the endothelial barrier function, and disruption of PF4-dependent complexes, ODSH targets fundamental drivers of tissue injury that are insufficiently addressed by existing therapies.

Our recent demonstration of ODSH’s protective effects in LPS-induced ALI [8] adds to a growing body of evidence supporting its role in pulmonary inflammation. Some clinical observations in AML and septic peritonitis further highlight its favorable safety profile and translational relevance.

Future work should prioritize several areas to advance ODSH toward clinical application: mechanistic resolution, e.g., delineating direct molecular interactions with HMGB1, PF4, selectins, and endothelial signaling pathways and distinguishing primary binding events from downstream effects; dose optimization and pharmacokinetics, e.g., defining therapeutic windows across organ systems and disease contexts; randomized clinical trials, e.g., evaluating ODSH in ALI/ARDS, CF exacerbations, sepsis, and oncology settings; comparative studies, e.g., assessing ODSH alongside other low-anticoagulant heparins to clarify structure–activity relationships and identify derivative-specific advantages; biomarker-guided approaches, e.g., identifying patient populations most likely to benefit based on HMGB1 levels, neutrophil elastase activity, PF4 profiles, or endothelial injury markers.

Taken together, ODSH represents a structurally refined glycosaminoglycan with the capacity to modulate conserved inflammatory and vascular pathways across diverse disease states. Continued investigation will determine its place within the evolving landscape of targeted anti-inflammatory and cytoprotective therapies.

## 9. Limitations and Outstanding Questions

Although ODSH has shown encouraging activity across multiple preclinical models, several limitations need to be addressed before its therapeutic potential can be fully defined. Mechanistic understanding remains incomplete. Existing studies demonstrate interactions with HMGB1, neutrophil elastase, PF4, and selectins, but the relative contribution of direct molecular binding versus indirect sequestration of extracellular mediators is not yet clear. Differentiating these mechanisms is essential for establishing structure–function relationships and predicting derivative-specific effects.

Pharmacokinetic data for ODSH are limited. Information on absorption, distribution, clearance, and dose–exposure relationships across organ systems is sparse, making it difficult to determine the optimal dosing strategies or therapeutic windows. This gap is particularly relevant for multifaceted diseases such as ALI/ARDS and sepsis, where timing and exposure may critically influence outcomes.

Comparative studies with other low-anticoagulant heparins are also lacking. Several desulfated derivatives exhibit overlapping anti-inflammatory or cytoprotective properties, yet systematic head-to-head evaluations have not been performed. Such comparisons are necessary to determine whether ODSH offers advantages over related compounds or whether multiple derivatives share similar mechanistic profiles.

Most evidence supporting ODSH comes from animal models. While pilot clinical observations in AML and septic peritonitis suggest acceptable safety, reliable human data are not yet available. Larger randomized double-blinded clinical trials are needed to evaluate efficacy, safety, and biomarker-guided patient selection in conditions such as ALI/ARDS, CF exacerbations, sepsis, and cancer. The potential for off-target interactions has not been fully explored. Heparin derivatives can bind a wide range of cationic proteins, and the broader consequences of these interactions, particularly during prolonged treatment or in multi-organ disease, require careful investigation.

Across the nonclinical literature, study designs vary substantially in species, dosing strategies, routes of administration, and reported endpoints. Most ODSH studies were designed to investigate mechanistic pathways rather than to meet regulatory toxicology reporting standards. As a result, detailed parameters required for formal reliability scoring frameworks such as Klimisch or ToxRTool (e.g., purity, stability, achieved concentrations, housing conditions) are not consistently available. Future work incorporating standardized pharmacokinetic, toxicology, immunogenicity, and dose–response assessments will be essential to support translational development.

Together, these limitations highlight the need for deeper mechanistic studies, comprehensive pharmacokinetic profiling, comparative analyses across heparin derivatives, and well-designed clinical trials to define the therapeutic role of ODSH.

## Figures and Tables

**Table 1:** Overview of main ODSH characteristics: structural features, mechanistic actions, and therapeutic rationale.

[A] Key Properties		References	PMID
(1) Selective 2-O and 3-O desulfation of heparin markedly reduces anti-X_a_/anti-IIa activity.		Qeios. 10.32388/ZCBHM9 [51,52]	NA
(2) Low-anticoagulant and non-anticoagulant profile		Fryer et al., 1997 [1]	9223556
(3) Quantifiable in biological samples		Buchanan et al., 2005 [4]	16213460
(4) Maintains high negative charge density, enabling binding to cationic proteins (PF4, HMGB1, chemokines, elastase)		Rao et al., 2010 [2]	20375277
[B] Key mechanisms/modes of action		References	PMID
(1) Inhibits p300 acetyltransferase activity, thereby limits HMGB1 secretion; binds extracellular HMGB1, disrupts HMGB1-RAGE and HMGB1-TLR4 signaling, further reduces downstream NF-κB activation and cytokine release. ODSH acts as a RAGE blocker.		Zheng et al., 2017 [53]	27585400
		Rao et al., 2010 [2]	20375277
		Wang et al., 2021 [5]	34271850
		Patil et al., 2026 [9]	NA
		Griffin et al., 2014 [6]	24325600
		Sefcik et al., 2011 [54]	NA
(2) Inhibit neutrophil elastase and reduce elastase-driven tissue injury as well as HMGB1 release.		Kummarapurugu et al., 2018 [32]	29903912
		Griffin et al., 2014 [6]	24325600
(3) Inhibit L-selectin, P-selectin, and E-selectin-mediated adhesion, decreasing leukocyte rolling, adhesion, and transmigration. Anti-inflammatory activity. Reduces endothelial activation.		Wang et al., 2002 [3]	12093896
		Silvestro et al., 1994 [20]	7824959
		Ramyaa Lakshmi et al., 2009 [21]	NA
		Lakshmi et al., 2010 [22]	20540094
(4) Block thrombin-induced endothelial permeability via PAR-dependent mechanisms and preserve junctional integrity (barrier-preserving).		Gonzales et al., 2014 [7]	24469066
		Gonzales et al., 2025 [8]	41008539
		Patil et al., 2026 [9]	NA
(5) Disrupts PF4/heparin multimolecular complexes, reduces HIT Ab binding, and blocks platelet activation.		Joglekar et al., 2010 [35]	NA
		Prechel et al., 2004 [37]	NA
(6) Relieve TFPIα mediated inhibition of prothrombinase, subtly shifting toward procoagulant activity without full anticoagulation. Reduced anticoagulant activity through altered TFPIα–prothrombinase regulation.		Wood et al., 2014 [55]	NA
		Wood et al., 2016 [56]	27301751
(7) Anti-oxidative and anti-ischemic actions: Non-anticoagulant heparins reduce oxidative stress, myocyte Na^+^/Ca^2+^ loading, as well as reperfusion injury.		Barry et al., 2010 [11]	19855066
[C] Therapeutic advantages	Rationale	References	PMID
(1) Minimal anticoagulant activity	Allows higher therapeutic dosing	Barry and Kennedy, 2011 [57]	21453252
(2) Potent anti-inflammatory; anti-protease effects	Inhibits RAGE, HMGB1, leukocyte adhesion, neutrophil elastase, and selectins; reduces tissue damage and cytokine storm	Voynow et al., 2020 [58]	32733248
		Sefcik et al., 2011 [54]	NA
(3) Endothelial barrier protection	Preserves VE-cadherin junctions, reduces LPS-induced permeability, oxidative stress, and edema, in lung/brain microvasculature	Gonzales et al., 2025 [8]	41008539
		Nagata et al., 2018 [12]	29373460
(4) Improved safety profile	Low bleeding risk at therapeutic doses; blocks HIT and platelet activation without provoking it	Rao et al., 2010 [2]	20375277
		Krauel et al., 2012 [59]	22049520
(5) Hematologic support; thrombocytopenia mitigation	Enhances count recovery after intensive AML therapy; mitigates chemotherapy/radiation-induced thrombocytopenia	Kovacsovics et al., 2014 [14]	NA
		Tkaczynski et al., 2018 [13]	29599195
(6) Oncologic; anti-metastatic potential	Low-anticoagulant heparins stop tumor cell–platelet interactions, P-selectin dependent adhesion, and metastatic dissemination.	Rao et al., 2011 [60]	NA
		Motta et al., 2024 [15]	38218079

Abbreviations: ODSH: 2-O, 3-O desulfated heparin; HMGB1: high mobility group box 1; PF4: platelet factor 4; RAGE: receptor for advanced glycation end products; TLR4: toll like receptor 4; NF-κB: nuclear factor kappa-light-chain-enhancer of activated B cells; PAR: protease-activated receptor; HIT: heparin-induced thrombocytopenia; Ab: antibody; TFPIα: tissue factor pathway inhibitor-alpha; VE-cadherin: vascular endothelial cadherin; LPS: lipopolysaccharide; AML: acute myeloid leukemia; NA: not available.

**Table 2:** Reported mechanisms and therapeutic effects of ODSH/low-anticoagulant desulfated heparin in disease contexts.

2-O, 3-O Desulfated Heparin/Low-Anticoagulant Desulfated Heparin			
Disease/Condition	Mechanism/Effects	References	PMID
(1) ALI/ARDS (pneumonia, LPS, smoke)	↓ vascular leak, ↓ lung edema, oxygenation ↑, preserves endothelial barrier via TLR4–MyD88–NF-κB modulation	Fryer et al., 1997 [1]	9223556
		Gonzales et al., 2014 [7]	24469066
		Gonzales et al., 2025 [8]	41008539
		Patil et al., 2026 [9]	NA
(2) Sepsis/endotoxemia/septic peritonitis	↓ organ injury, ↓ leukocyte adhesion, ↓ HMGB1–RAGE signaling; demonstrated safety across ascending dose levels in septic peritonitis (veterinary model)	Rao et al., 2010 [2]	20375277
		Mauro et al., 2022 [17]	35498738
(3) *Pseudomonas pneumonia*	↓ lung injury, ↑ bacterial clearance, improves survival with partially desulfated heparin	Sharma et al., 2014 [69]	24099632
(4) CF lung disease	↓ lung injury; ↑ bacterial clearance, restores HMGB1-compromised macrophage function, attenuates neutrophil elastases and HMGB1 secretion	Griffin et al., 2014 [6]	24325600
		Voynow et al., 2020 [58]	32733248
		Wang et al., 2021 [5]	34271850
(5) I/R injury (brain, heart)	↓ leukocyte adhesion, ↓ oxidative stress, ↓ infarct size and edema; improves neurologic outcome after cerebral I/R	Mocco et al., 2007 [10]	18162910
		Barry et al., 2010 [11]	19855066
		Barry and Kennedy, 2011 [57]	21453252
(6) TBI	↑ cognitive recovery, ↓ leukocyte mobilization, ↓ brain edema	Nagata et al., 2018 [12]	29373460
(7) Metastatic cancer (NSCLC, pancreatic, melanoma)	Mitigates tumor cell–platelet association, metastatic spread and P-selectin-mediated adhesion; in pancreatic models enhances gemcitabine efficacy	Gao et al., 2006 [16]	16331491
		Rao et al., 2011 [60]	NA
		Marcus et al., 2012 [46]	NA
		Sigal et al., 2013 [73]	NA
(8) Chemotherapy-induced/radiation-induced thrombocytopenia	Inhibits thrombocytopenia, promotes platelet recovery after chemo-radiation and intensive (high dose) AML therapy	Kovacsovics et al., 2014 [14]	NA
		Tkaczynski et al., 2018 [13]	29599195
(9) Post-subarachnoid hemorrhage brain injury	Heparin and derivatives are considered as multimodal therapy targeting inflammation, microthrombosis, and vasospasm.	Hayman et al., 2017 [74]	28468328
(10) Radiation combined burn injury	Evaluated as a countermeasure; dosing studies suggest protective, anti-inflammatory potential	Islam et al., 2015 [75]	NA
(11) HIT/PF4 mediated disorders	Attenuates HIT antibody-induced platelet activation, ↓ HIT risk, disrupts PF4/heparin complexes	Prechel et al., 2004 [37]	NA
		Joglekar et al., 2010 [35]	NA
		Joglekar et al., 2012 [36]	22318669
		Krauel et al., 2012 [59]	22049520
		Jouni et al., 2016 [76]	26423467
(12) Rosacea/cutaneous inflammation	Disrupt RAGE ligands and ↓ cutaneous inflammation (Novel sulfated polysaccharides (related low-anticoagulant derivatives))	Zhang et al., 2011 [77]	21347371

↓ decrease; ↑ increase. Abbreviations: ODSH: 2-O, 3-O desulfated heparin; ALI: acute lung injury; ARDS: acute respiratory distress syndrome; LPS: lipopolysaccharide; TLR4: toll-like receptor 4; MyD88: myeloid differentiation primary response protein 88; NF-κB: nuclear factor kappa-light-chain-enhancer of activated B cells; HMGB1: high mobility group box 1; RAGE: receptor for advanced glycation end products; CF: cystic fibrosis; I/R: ischemia/reperfusion; TBI: traumatic brain injury; NSCLC: non-small cell lung cancer; AML: acute myeloid leukemia; HIT: heparin-induced thrombocytopenia; PF4: platelet factor 4; NA: not available.

## Data Availability

No new data were created or analyzed in this study. Data sharing is not applicable to this article.

## Abbreviations

The following abbreviations are used in this manuscript:

Ab	Antibody
ALI	Acute Lung Injury
AML	Acute Myeloid Leukemia
ARDS	Acute Respiratory Distress Syndrome
BBB	Blood–Brain Barrier
CF	Cystic Fibrosis
GAGs	Glycosaminoglycans
ERK	Extracellular Signal-Regulated Kinase
FGF2	Fibroblast Growth Factor 2
FGFR1	Fibroblast Growth Factor Receptor 1
HIT	Heparin-Induced Thrombocytopenia
HMGB1	High Mobility Group Box 1
I/R	Ischemia–Reperfusion
LPS	Lipopolysaccharide
NF-κB	Nuclear Factor kappa-light-chain-enhancer of activated B cells
ODSH	2-O, 3-O-Desulfated Heparin
PAR	Protease-Activated Receptor
PF4	Platelet Factor 4
RAGE	Receptor for Advanced Glycation End Products
ROS	Reactive Oxygen Species
TBI	Traumatic Brain Injury
TF	Tissue Factor
TFPIα	Tissue Factor Pathway Inhibitor-α
TLR4	Toll-Like Receptor 4
VE-cadherin	Vascular Endothelial Cadherin
